# Human Muscle Satellite Cells as Targets of Chikungunya Virus Infection

**DOI:** 10.1371/journal.pone.0000527

**Published:** 2007-06-13

**Authors:** Simona Ozden, Michel Huerre, Jean-Pierre Riviere, Lark L. Coffey, Philippe V. Afonso, Vincent Mouly, Jean de Monredon, Jean-Christophe Roger, Mohamed El Amrani, Jean-Luc Yvin, Marie-Christine Jaffar, Marie-Pascale Frenkiel, Marion Sourisseau, Olivier Schwartz, Gillian Butler-Browne, Philippe Desprès, Antoine Gessain, Pierre-Emmanuel Ceccaldi

**Affiliations:** 1 Unité Epidémiologie et Physiopathologie des Virus Oncogènes-CNRS URA1930, Institut Pasteur, Paris, France; 2 Unité Recherche et Expertise Histotechnologie et Pathologie, Institut Pasteur, Paris, France; 3 Service d'Anatomopathologie, CHD Félix Guyon, Saint-Denis de la Réunion, France; 4 Unité Interactions Moléculaires Flavivirus-Hôtes, Institut Pasteur, Paris, France; 5 Inserm U787-Université Pierre et Marie Curie-Institut de Myologie, Pitié Salpêtrière, Paris, France; 6 Service de Neurologie, CHD Félix Guyon, Saint-Denis de la Réunion, France; 7 Service de Médecine Interne, CHD Félix Guyon, Saint-Denis de la Réunion, France; 8 Laboratoire de Biologie, CHD Félix Guyon, Saint-Denis de la Réunion, France; 9 Unité Virus et Immunité, Institut Pasteur, Paris, France; AIDS Research Center, Chinese Academy of Medical Sciences and Peking Union Medical College, China

## Abstract

**Background:**

Chikungunya (CHIK) virus is a mosquito-transmitted alphavirus that causes in humans an acute infection characterised by fever, polyarthralgia, head-ache, and myalgia. Since 2005, the emergence of CHIK virus was associated with an unprecedented magnitude outbreak of CHIK disease in the Indian Ocean. Clinically, this outbreak was characterized by invalidating poly-arthralgia, with myalgia being reported in 97.7% of cases. Since the cellular targets of CHIK virus in humans are unknown, we studied the pathogenic events and targets of CHIK infection in skeletal muscle.

**Methodology/Principal Findings:**

Immunohistology on muscle biopsies from two CHIK virus-infected patients with myositic syndrome showed that viral antigens were found exclusively inside skeletal muscle progenitor cells (designed as satelllite cells), and not in muscle fibers. To evaluate the ability of CHIK virus to replicate in human satellite cells, we assessed virus infection on primary human muscle cells; viral growth was observed in CHIK virus-infected satellite cells with a cytopathic effect, whereas myotubes were essentially refractory to infection.

**Conclusions/Significance:**

This report provides new insights into CHIK virus pathogenesis, since it is the first to identify a cellular target of CHIK virus in humans and to report a selective infection of muscle satellite cells by a viral agent in humans.

## Introduction

Chikungunya virus (CHIKV) is a moquito-transmitted alphavirus belonging to the family *Togaviridae*, which was first reported in 1952 in Tanganyika. It is responsible for an acute infection of abrupt onset, characterised by high fever, arthralgia, myalgia, head-ache, chills, photophobia and rash [1]. The symptoms are generally of short duration (one week) and recovery is often complete, although some patients have recurrent episodes for several weeks after infection[1], [2]. This virus is endemic in Africa, India and South-East Asia and is transmitted by Aedes mosquitoes through an urban or sylvatic transmission cycle. CHIK virus has massively emerged in the Indian Ocean, with more than 200.000 cases being reported in the Réunion island by March 2006 [3], and more than 1.25 million suspected cases in India between February and August, 2006 [4]. More recently, imported cases of CHIK virus infection have been observed in France, Germany, Switzerland and Norway [5]. From a clinical point of view, the Réunion outbreak was characterized by an atypical magnitude and virulence, with painful and invalidating poly-arthralgia, and with myalgia being reported as a major clinical symptom (97.7% of cases) [6]. A recent clinical study has reported an occurrence of rhabdomyolysis with high creatine phosphokinase (CPK) [7]. Little is known about the pathophysiological mechanisms of CHIK virus infection, and especially about the cellular targets of CHIK virus. We investigated the possible involvement of muscle cells in the CHIK pathogenesis, both by an *ex-vivo* approach in muscle biopsies from two infected patients with a myositic syndrome, and *in vitro* on cultures of human muscle satellite cells, that can be differentiated into myotubes.

## Materials and Methods

### Ex vivo study:

Two muscle biopsies were studied. The first muscle biopsy was obtained from Patient #1 during the CHIK virus epidemic outbreak in the Reunion Island in 2005. This patient, who was 67-year old, presented signs of renal failure, hypertension, and ethylism. He was admitted to hospital with a classical clinical picture of CHIK virus infection: polyarthtralgia, fever, and myalgia. In addition, signs of rhabdomyolysis, in the absence of trauma, were also reported (CPK 41600 IU/mL; myoglobin 8300 IU/mL). RT-PCR for CHIK virus was negative in the serum, and low level of specific CHIK virus IgM was detected (0.5 IU/mL). Serology for dengue virus was in the limit range (IgM: 1.9 IU/mL), and was negative for Epstein Barr virus and hepatitis viruses. Muscle biopsy was performed on the quadriceps muscle, for anatomopathological diagnosis purpose (with institutional approval of the F. Guyon hospital, La Réunion), during the acute phase of the illness (around ten days after the beginning of the crisis). During the following weeks, the patient recovered.

Concerning the second biopsy, Patient#2 is a 71-year old woman who complained in January, 2006, of headaches, arthralgia and a rash. Around three months later, she was admitted to hospital with a classical clinical picture of CHIK virus infection, including fever, headache, joint pain, and myalgia, accompanied by muscle weakness in the four limbs, with a myogenic electromyogram without fibrillations. Serology was negative for hepatitis B and C viruses, HIV, HTLV-1 and 2, dengue virus, leptospirosis. At this stage, CHIKV specific IgG were at 40.9 and IgM: 1.1 I.U./mL, and 39.3 and <1 I.U./mL respectively, one month later. Muscle biopsy was performed for anatomopathological diagnosis purpose on the quadriceps muscle during this recurrent phase of the disease.

### Immunocytochemistry

Immunocytochemical analysis was performed on cell cultures as well as sections of the muscle biopsy both by indirect immunofluorescence or immunoperoxidase. Cultures were fixed in acetone for 10 min. Immunocytochemistry was performed after incubation for 30 minutes with 10% normal goat serum diluted in PBS, to avoid nonspecific antibody binding. Two different HMAFs, produced at the Pasteur Institute (Paris), were used as primary antibodies; one HMAF “#1”, previously reported[8] was used at a dilution of 1/1000, whereas the second (#2) was used in the concentration range of 1/1000-1/1600. In other experiments, a monoclonal anti-Semliki virus directed against a conserved region of the alphavirus nucleocapsid protein, diluted at 1/50 was used [9]. HMAFs against French neurotropic virus strain of yellow fever virus, West Nile virus (IS-98-ST1 strain) or Hawai strain of dengue type-1 virus were used as primary antibodies for negative controls as previously described [8], [10]. Other antibodies that were purchased from Dako (Glostrup, Denmark)(mouse monoclonal anti-CD56, -CD8, -CD68 and –CD20, “ready-to-use Envision”), from Neomarker (Fremont, CA, USA) (rabbit anti-CD3), or from Sigma (St Louis, MI, USA) (rabbit anti-laminin), and were used according to manufacturer's instructions.

For immunofluorescence, specific secondary antibodies coupled with fluorescein (Vector Laboratories, CA, USA) were used according to the manufacturer's instructions. Antibodies were incubated in 0.05% saponin and 10% normal goat serum over night at 4°C or for 1 h. at room temperature. After washes, preparations were mounted in Vectashield medium (Vector laboratories, CA, USA). In all experiments, viral proteins were also visualized by immunoperoxydase on fixed cell cultures using Peroxidase ImmPRESS Reagent Kit anti-Mouse immunoglobulins (Vector Laboratories, CA, USA) and DAB Liquid Substrate System according to the manufacturer's instructions.

For paraffin-embedded sections obtained from quadriceps muscle biopsies, after paraffine removal, sections were rehydrated and incubated for 30 minutes with 10% normal goat serum and processed for viral antigens detection. Sections of muscles from a non-infected patient and from a HTLV-1-infected patient with a myositic syndrome, were used as negative controls, as reported in a recent paper [11].

### Cells and culture conditions:

Satellite cells (i.e. myogenic precursor cells that persist in postnatal and adult muscle) were originally isolated from the quadriceps of a 5-day-old infant as previously described (“CHQ5B”cells) [12]. Two other primary muscle satellite cell cultures (so called “KM162C14Q” and “KM155C25”) from two donors, 14 and 25 year old, respectively, were also used in one experiment to test the susceptibility to CHIKV infection. Cells were provided by the AFM Tissue Bank (Paris, France); informed consent was obtained from all patients prior to the tissue being donated to the tisue bank, in accordance with the French legislation on bioethics, and with institutional approval of the Pitié-Salpêtrière Hospital (Paris, France). The cells cultivated in Ham's F-10 supplemented with 50 µg/ml of gentamycin and 20% fetal calf serum were trypsinized when they reached half confluency. Differentiation of satellite cells into myotubes was induced for three to five days in DMEM medium supplemented with 100 µg/ml transferrin, 10 µg/ml insulin and gentamycin [12].

### Virus infection:

Muscle satellite cells were grown on coverslips in 24-well plates, and, for some cultures, differentiated for three to five days into myotubes. Muscle satellite cells or myotube cultures were then infected with two different isolates of CHIK virus or vehicle alone. Both isolates, 05-115 and 06-49 were obtained during the 2005–2006 Reunion outbreak, through a passage on C6/36 mosquitoe cells as described previously [8]. Isolate 05-115 has been described as the ancestral genotype of the Reunion outbreak (Genotype 1), whereas isolate 06-049 (Genotype 4) has been described to have arisen after three distinct synonymous substitutions, and one amino-acid change in E1 protein (Ala–>Val; site 226) [8]. For infection experiments, muscle cells (proliferating and terminally differentiated myotubes) were exposed for 2 h. at 37°C in medium containing 1% FCS to about 10 ffu/cell of CHIK virus isolates. Cultures were kept for 24, 36, 48, and 72h. (according to experiments) in Ham's F-10 medium supplemented with 50 µg/ml of gentamycin and 2% fetal calf serum. Control experiments were performed using mock-infected muscle cell cultures (satellite cells or myotubes).

## Results

### Ex-vivo studies: clinical cases

**Figure 1:**
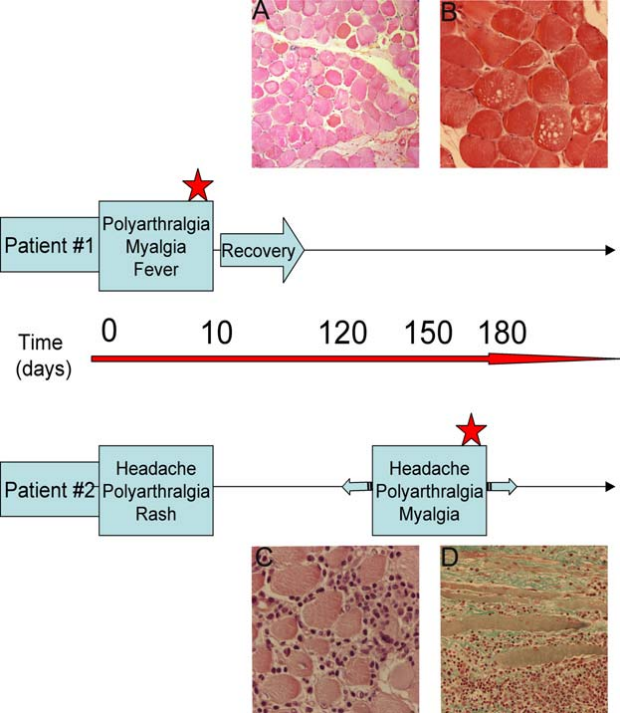
Time-course and histopathological data concerning CHIK virus-infected patients #1 and #2. Patient #1, 67 year old, (from whom a quadriceps biopsy was obtained during the CHIK virus epidemic outbreak in the Reunion Island) presented a classical clinical picture of CHIK virus infection: polyarthtralgia, fever, and myalgia. In addition, signs of rhabdomyolysis were reported (creatine phosphokinase 41600 IU/mL). The muscle biopsy was performed on the quadriceps muscle, for pathological diagnosis during the acute phase of the illness (red star). During the following weeks, the patient recovered. Patient#2 is a 71 year old woman who complained in January, 2006, of headaches, arthralgia and a rash; around three months later, she was admitted to hospital with a classical clinical picture of CHIK virus infection, including fever, headaches, joint pain, and myalgia. A biopsy was performed in the quadriceps muscle during this recurrent phase of the disease (red star). 1A: A section from the muscle biopsy of patient #1. Hematoxylin-eosin staining. No important cellular infiltrates were observed. Atrophy and necrosis of muscle fibers could be seen, as well as central nuclei (arrow). (magnification: ×60). 1B: A section from the muscle biopsy of patient #1. Hematoxylin-eosin staining. Vacuolization of muscle fibers were detected. (magnification: ×140). 1C, D: Sections from the muscle biopsy of patient #2 showing an important mononuclear infiltration in transversal (C) and longitudinal sections (D) stained by hematoxylin-eosin (C) and Masson's trichrome (D); an important amount of fibrosis is also observed (D); (magnification:C: ×140; D: ×80)

In order to identify the possible cellular targets of CHIK virus within the muscle *in vivo*, biopsies from two infected patients with a myositic syndrome were analyzed (Fig.1A). Patient#1 is a 67-year old man, with a classical clinical picture of CHIK virus infection, including polyarthralgia, headache and myalgia. In addition, signs of renal failure and rhabdomyolysis were observed (CPK 41.600 IU/mL). A quadriceps biopsy was performed around ten days after the beginning of the symptoms, for pathological diagnosis, at the end of the acute phase of the disease. In the following weeks, patient#1 recovered. Patient#2 is a 71-year old woman who developed in January 2006 a classical CHIK virus syndrome including headache, arthralgia and rash. Around three months later, she was admitted to hospital with reccurrency of classical CHIK virus infection clinical picture, included fever, headache, joint pain, and myalgia. A quadriceps muscle biopsy was then performed for pathological diagnosis. This corresponds to a recurrent episode of the disease as compared to patient#1 (Fig. 1A).

### Ex-vivo study: histology

A histological study of these muscle biopsies revealed two different features of pathological changes. In sections from patient#1 biopsy, atrophy and necrosis of scattered muscle fibers (Fig. 1A), with a limited number of infiltrating cells were observed. Vacuolization of muscle fibers could also be seen (Fig. 1B). In contrast, sections from patient#2 biopsy indicated the presence of extensive interstitial mixed acute and chronic inflammation (Fig. 1C), including interstitial polymorphonuclear cells, lymphocytes and histiocytes. Large areas of necrosis and collagenosis were also observed, as shown in Fig.1D.

### Ex-vivo study: immunohistochemistry

**Figure 2:**
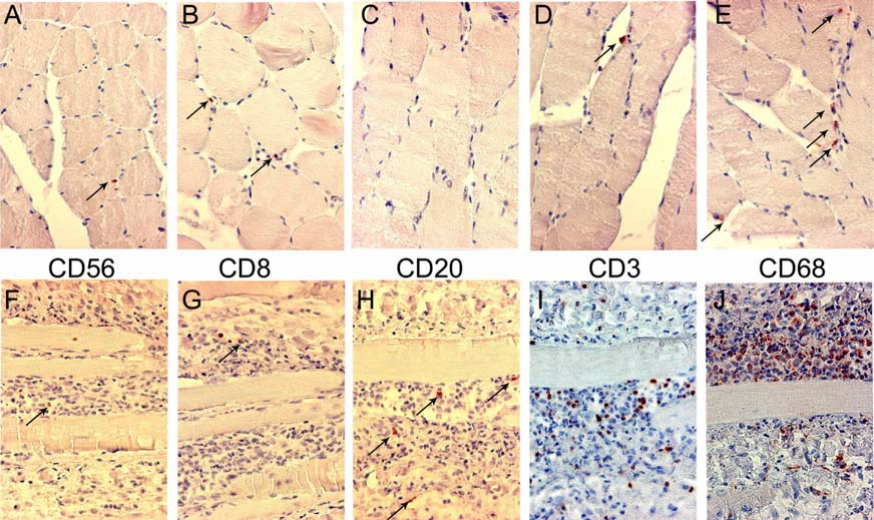
Identification of infiltrating cells by immunocytochemistry. Muscle sections from patient#1 (A to E)) or patient#2 (F to J) werre processed as described in material and methods for immunocytochemistry (immunoperoxydase detection) for the following antigens: CD56 (mouse monoclonal antibody; Dako; A,E); CD8 (mouse monoclonal antibody; Dako; B,G); CD20 (mouse monoclonal antibody; Dako; C,H); CD3 (Neomarker; D,I); CD68 (mouse monoclonal antibody; Dako; E,J). In patient#1 (A to E), the few infiltrating cells were mostly detected as immunoreactive for CD68 and CD3. In patient#2, the massive infiltrates were mainly composed of CD68 and CD3 immunoreactive cells. Counterstain: henatoxylin-eosin. Immunoreactive cells (red-brown color) are indicated by arrows, except in I and J (too numerous). Magnification: ×170.

Infiltrating cells were identified by immunohistochemistry. In muscle sections from patient#1, the rare infiltrating cells that were seen were mostly immunoreactive for CD3 or CD8, or CD68 (Fig. 2 D,B,E, respectively), indicating the presence of T cells (suppressor/cytotoxic subpopulation) and macrophages within the infiltrate; rare or no cells immunoreactive for CD56 (natural killer cells) or CD20 (B cells) were also observed (Fig. 2A,C, respectively).

In muscle sections from patient#2, the numerous infiltrating cells were found immunoreactive mostly for CD68 (macrophages) (Fig. 2J) and for CD3 (T cells)(Fig. 2I). A few cells immunoreactive for CD56, CD8 or CD20 could be seen (Fig. 2. F,G,H, respectively). Taken together these patterns have no specificity and are consistent with a diagnosis of viral myositis.

### Ex-vivo study: viral antigens detection by immunohistochemistry

**Figure 3:**
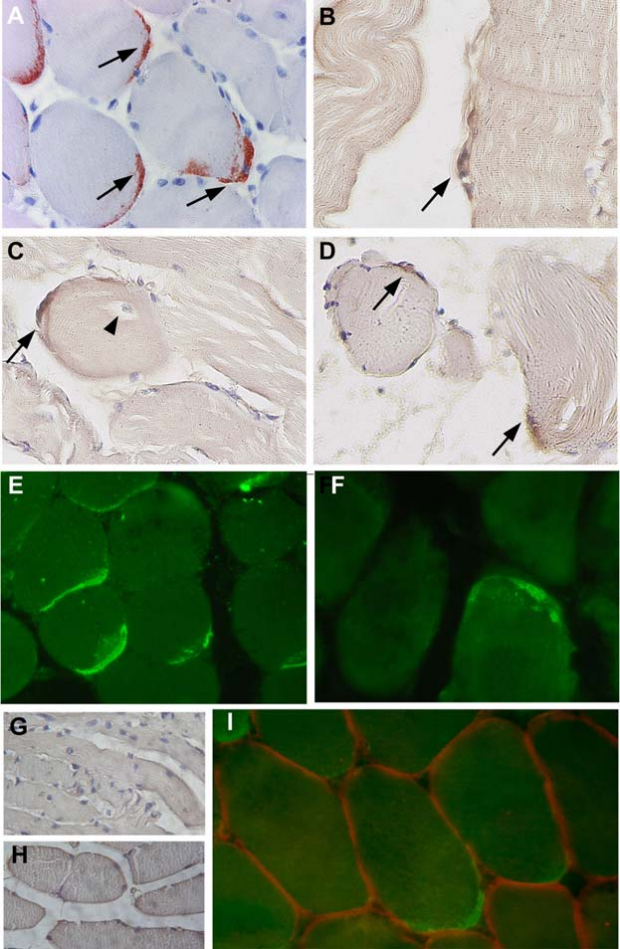
Immunocytochemical detection of CHIK virus antigens in a section of the muscle biopsy from patients #1 and #2. Detection of CHIK virus antigens was performed on paraffin embedded sections (indirect immunofluorescence and immunoperoxydase) A–D: Detection of CHIK virus in muscle biopsy sections from patient #1 (immunoperoxydase) (Fig 1A: AEC as substrate; Fig. B,C,D: DAB as substrate). Immunoreactivity is detected on fusiform curved shaped cells located at the periphery of the myotubes, either as multiple cells per microscopic field (A,D), or as single cell per field (B,C). Some immunoreactive cells (arrows) were detected at the periphery of muscle fibers with central nuclei (C, arrowhead). E–F: Detection of CHIK virus in muscle biopsy sections from patient #1 (E) and #2 (F) using the immunofluorescence technique. Whereas the muscle biopsy of patient #1 numerous cells exhibited immunoreactivity (E), at the periphery of myotubes, as assessed by the (immunoperoxydase technique), very rare immunoreactive cells could be detected in sections of the muscle biopsy from patient #2 (F). G: West Nile virus (IS-98-ST1 srtrain) used as the primary antibody (muscle section from patient#1); no staining was observed; similar results were obtained with sera against yellow fever virus, or dengue type-1 virus (data not shown). G,H: sections from a non-infected patient (G) or from a HTLV-1-infected patient with myositic syndrome[11] (H) used as controls; no significant immunoreactivity detected. I: double labeling by immunofluorescence of CHIK virus antigens (green staining; HMAFs anti CHIK virus #1) and laminin (red staining; rabbit anti-laminin polyclonal antibody). Note the CHIK virus immunoreactive cells are located beneath the basal lamina. (magnification: ×300(A,C,D,E,F); ×400(B); ×420 (I); ×100 (G,H)).

In the biopsy of patient#1, CHIK viral antigens could be detected by immunohistochemistry (using anti-CHIK virus hyperimmune mouse ascitic fluids [8], HMAFs) at the periphery of muscle fibers, either as single cells (Fig. 3B, C) or groups of immunoreactive cells (Fig. 3A, D). In some cases, positive cells were found surrounding muscle fibers with central nuclei (Fig. 3C). Similar results were obtained with two different ascitic fluids, by immunofluorescence or immunoperoxydase staining (Fig. 3E). Importantly, all the immunoreactive cells exhibited a fusiform curved shape reminiscent of satellite cells. This was confirmed by their location beneath the basal lamina, as shown by double labeling for CHIK viral antigen and laminin (Fig. 3I), or by double labeling of satellite cells for CHIK viral antigen and Neural Cell Adhesion Molecule, which is an additional criterion to identify satellite cells (data not shown). Muscle fibers were never found positive for CHIK viral antigen, in all the sections examined (Fig. 3A–E). Moreover, we did not find any labeling of infiltrating cells, nor endothelial cells. CHIK viral antigens could also be visualized in muscle biopsy sections from patient#2, albeit at a lower magnitude (Fig. 3F). Given that the biopsy from patient#2 was performed during a recurrent phase of CHIK disease, it might be predicted that satellite cells are still subjected to CHIK virus replication past the initial stages of virus infection.

We further checked the specificity of the labeling by using as controls ascitic fluids against yellow fever, West Nile or dengue type-1 viruses[8], [10]. No immunoreactive cells were detected (data not shown). In addition, no signal could be seen in biopsies samples from three different non-infected control individuals (Fig 2G, and data not shown) and from a HTLV-1-infected patient with myositic syndrome[11] using anti-CHIK virus HMAF as primary antibody (Fig. 2H).

### In vitro study

**Figure 4:**
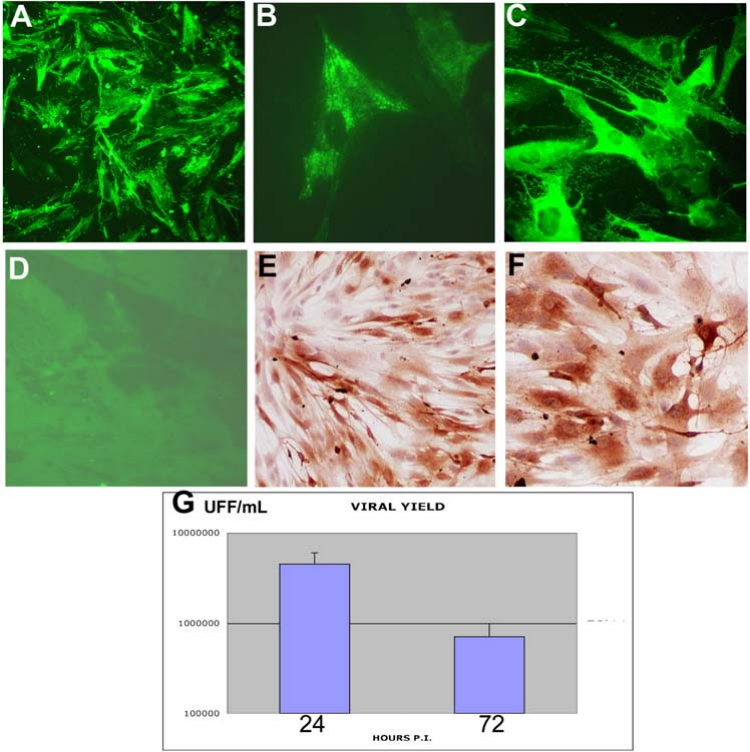
Infection of cultured human muscle satellite cells by CHIK virus. Human muscle satellite cells were isolated from the quadriceps (surgical wasting)as previously described [12]; They were grown on coverslips and, once differentiated into mytubes or not, infected with two different CHIK virus isolates[8] (isolates 05-115 and 06-049) or vehicle alone. For infection experiments, muscle cells (satellites and myotubes) were exposed for 2 h. at 37°C in medium containing 1% FCS to about 1 to 10 pfu/cell of CHIK virus isolates 06-049 and 05-115 . Cultures were kept for 24, 36, 48, and 72 h. (according to experiments) in Ham's F-10 medium supplemented with 2% fetal calf serum. Immunocytochemical analysis was performed as described in Fig. 2, both by indirect immunofluorescence or immunoperoxydase, after acetone fixation. A: Detection by immunofluorescence of CHIK virus antigens on satellite cells grown for 24 h. after CHIK virus infection (m.o.i. 10). HMAF anti-CHIK virus #1; (magnification:×250). B: Detection by immunofluorescence of CHIKV antigens on satellite cells grown for 24 h. after CHIK virus infection (m.o.i. 10), using a monoclonal antibody against alphavirus nucleocapsid[9]. (magnification: ×1000). C: Detection by immunofluorescence of CHIK virus antigens on satellite cells grown for 48 h. after CHIK virus infection (m.o.i. 10). HMAF anti-CHIK virus #1; (magnification: ×400). D: Absence of infection of myotubes, 24 h. after CHIK virus infection (m.o.i. 10), using HMAF anti-CHIK virus as primary antibody. (magnification: ×200). E and F: Detection by immunoperoxydase of CHIK virus antigens on satellite cells (from two different donors) grown for 36 h. after CHIK virus infection (m.o.i. 10). HMAF anti-CHIK virus #1; (magnification: ×200(E), ×400(F)). G: viral yielding of satellite cells from donor #1 at 24 and 72 h. post-infection by CHIK virus. Analysis of viral yielding was performed by plaque titration on cultured mosquitoe cells as previously described[8] and is expressed as UFF/mL.

To examine whether CHIK virus exhibits a particular tropism for human satellite cells, we performed an *in vitro* approach, according to a previous model developed in the laboratory (Ozden et al., 2005). Human primary muscle cell cultures, differentiated or not into myotubes, were inoculated with low-passaged CHIK virus strains obtained during the 2005–2006 Reunion outbreak[8], at low to high (1 or 10) multiplicity of infection (m.o.i.). At 24 h. p.i., cultures of satellite cells were found to be infected by both strains (Fig. 4A, and data not shown). Viral antigens could also be detected by immunofluorescence using a monoclonal antibody directed against a conserved region of the alphavirus nucleocapsid[9], with the signal being mainly located in endoplasmic reticulum-like structures (Fig. 4B). No signal was detected on mock-infected cultures, or on CHIK virus-infected cultures with an irrelevant antibody (data not shown). Interestingly, on differentiated myotube cultures, no immunoreactivity could be observed, using anti-CHIK virus HMAF or nucleocapsid monoclonal antibody (data not shown and Fig. 4D). Similar results were obtained with muscle cells from two other donors, at 24 h. as well as at 36 h. p.i. (Fig. 4E,F). At 48 h. p.i., around 100% of cells were infected, whatever the viral isolate or the donor (see Fig. 4C). In all cases, between 24 and 72 h. p.i. a strong cytopathic effect was observed, and at 96 h. post-infection almost all infected cells had died (data not shown). Analysis of viral yields on plaque titration revealed that the viral yield reached a value of 3 to 6 10^6^ FFU/mL at 24 h. post-infection, and decreased to 4–9.5 10^5^ FFU/mL at 72 h. post-infection, in parallel with the cell death (Fig 4G).

## Discussion

Following the unprecedented magnitude outbreak of CHIK virus in La Réunion[3], [4], [13] in 2005, the present work constitutes the first anatomopathological report of myositis associated with CHIK virus infection in humans. Although the expression patterns of the few (patient#1) or numerous (patient#2) infiltrating cells is not specific and is consistent with a diagnosis of viral myositis such as Coxsackie virus[14], these results constitute the first observation of a selective viral infection of muscle satellite cells in humans. Muscle satellite cells are myogenic precursor cells that persist in mature skeletal muscle as quiescent cells [15]. They are located under the basal lamina of the muscle fiber and contribute myonuclei to growing muscle fibers by cell fusion. They are considered as the main, if not only, cell type responsible for postnatal muscle growth and repair [16]. Because CHIK virus-infected cells have been observed three to four months after the acute crisis, we hypothesize that infection of precursor cells may have pathological consequences on long-term muscle physiology in patients. Consistent with this, susceptibility of satellite cells might be crucial for the physiopathology of CHIK virus infection in humans with a possible persistence of virus on muscle tissue leading to recurrent myalgia. Another conclusion of these observations is that sections from patient#2 showed more infiltrating cells (mainly macrophages and T cells) than patient#1, but less satellite cell immunoreactive for CHIK virus antigens. Since patient#2 corresponds to a more chronic stage of the disease, it can be hypothesized that some CHIK virus-infected satellite cells could be eliminated, either by a direct cytopathic effect or through the immune response.

The pathogenesis of CHIK virus infection in humans is still poorly understood. In a recent study, CHIK virus isolates from La Reunion outbreak were found to infect *in vitro* human epithelial and endothelial cells, primary fibroblasts, and, to a lower extent monocyte-derived macrophages, but not primary lymphocytes and monocytes, nor monocyte-derived dendritic cells[17] Muscle cells have been proposed to be target cells for alphavirus infection[18], [19], [20], [21], [22]. Muscle necrosis has been observed during infection of both trout and salmon by alphaviruses such as Sleeping Disease Virus[22]. Mayaro virus has been reported to induce myalgias in humans, and muscle necrosis in animal models[23]. Besides these alterations, alphavirus infection of muscle has also been reported, as Ross River Virus in a mouse model[20], [24], Getah virus that infects and induces degenerative changes in myofibers[18], or Semliki Forest Virus that infects murine muscle cultures[25]. However, these studies were either performed on animal models, or only based on clinical observations in man, and the cellular target of virus infection was either not identified within the muscle, or identified as muscle fibers and/or infiltrating cells.

Our experimental model based on CHIK virus infection of human satellite cells may be useful to gain insights into the regulated expression of cell surface molecules which function as CHIK virus attachment factors or receptors. Indeed, such a regulation of a receptor to an alphavirus has already been reported, on mouse brain cells that express differentially a Sindbis virus receptor during development[26].

The ability of CHIK virus to selectively infect progenitor cells involved in muscle repair provides new keys to our understanding of the long term evolution of pathogen-induced myopathies through necrosis, defects in muscle regeneration, and possibly the role of a viral reservoir in recurrent crises. In this context, further observations especially in patients suffering from recurrent crises of myalgia would provide precious data on chronic infections and necrosis/regeneration processes within the muscle, and would help to assess the relevance of persistently infected muscle cells in CHIK virus-infected patients.
